# Diaqua­bis(3,7-dichloro­quinoline-8-carboxyl­ato)zinc(II) monohydrate

**DOI:** 10.1107/S1600536808025671

**Published:** 2008-08-16

**Authors:** Li-Tao An, Jian Zhou, Jian-Feng Zhou, Min Xia

**Affiliations:** a Jiangsu Key Laboratory for Chemistry of Low-dimensional Materials, Department of Chemistry, Huaiyin Teachers College, Huaian 223300, Jiangsu Province, People’s Republic of China; b Department of Chemistry and Biology, Yulin Normal University, Yulin 537000, People’s Republic of China

## Abstract

In the title compound, [Zn(C_10_H_4_Cl_2_NO_2_)_2_(H_2_O)_2_]·H_2_O, the Zn atom has a distorted square-pyramidal geometry comprising two O atoms and one N atom from two distinct 3,7-dichloro­quinoline-8-carboxyl­ate ligands, and two water mol­ecules. The free water mol­ecules are involved in inter­molecular O—H⋯O hydrogen bonding with the coordinated water mol­ecules and carboxyl­ate O atoms, to give a one-dimensional helical chain along the [100] direction.

## Related literature

For related literature, see: Adnan *et al.* (2003 ▶); Che *et al.* (2005 ▶); Chen *et al.* (2001 ▶); Li *et al.* (2008 ▶); Lumme *et al.* (1984 ▶); Yang *et al.* (2005 ▶); Zhang *et al.* (2007 ▶). 
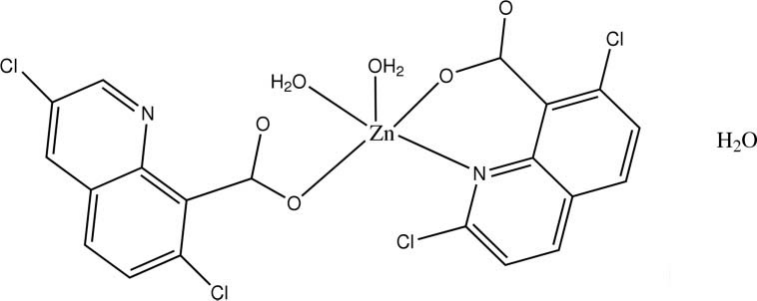


## Experimental

### 

#### Crystal data

[Zn(C_10_H_4_Cl_2_NO_2_)_2_(H_2_O)_2_]·H_2_O
*M* *_r_* = 601.50
Triclinic, 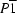

*a* = 6.8678 (3) Å
*b* = 12.6996 (5) Å
*c* = 12.9317 (5) Å
α = 87.572 (1)°
β = 82.893 (1)°
γ = 82.255 (1)°
*V* = 1108.63 (8) Å^3^
*Z* = 2
Mo *K*α radiation
μ = 1.64 mm^−1^
*T* = 296 (2) K
0.21 × 0.17 × 0.15 mm

#### Data collection

Rigaku Mercury diffractometer
Absorption correction: multi-scan (*CrystalClear*; Rigaku/MSC, 2001 ▶) *T*_min_ = 0.725, *T*_max_ = 0.791
14022 measured reflections
4308 independent reflections
3099 reflections with *I* > 2σ(*I*)
*R*_int_ = 0.080

#### Refinement

*R*[*F* ^2^ > 2σ(*F* ^2^)] = 0.041
*wR*(*F* ^2^) = 0.094
*S* = 0.97
4308 reflections
307 parameters
H-atom parameters constrained
Δρ_max_ = 1.20 e Å^−3^
Δρ_min_ = −0.80 e Å^−3^

### 

Data collection: *CrystalClear* (Rigaku/MSC, 2001 ▶); cell refinement: *CrystalClear*; data reduction: *CrystalStructure* (Rigaku/MSC, 2004 ▶); program(s) used to solve structure: *SHELXS97* (Sheldrick, 2008 ▶); program(s) used to refine structure: *SHELXL97* (Sheldrick, 2008 ▶); molecular graphics: *ORTEPII* (Johnson, 1976 ▶); software used to prepare material for publication: *SHELXL97*.

## Supplementary Material

Crystal structure: contains datablocks global, I. DOI: 10.1107/S1600536808025671/sg2255sup1.cif

Structure factors: contains datablocks I. DOI: 10.1107/S1600536808025671/sg2255Isup2.hkl

Additional supplementary materials:  crystallographic information; 3D view; checkCIF report

## Figures and Tables

**Table 1:** Hydrogen-bond geometry (Å, °)

*D*—H⋯*A*	*D*—H	H⋯*A*	*D*⋯*A*	*D*—H⋯*A*
O5—H5*A*⋯O1^i^	0.85	2.05	2.889 (4)	166
O5—H5*B*⋯N2	0.86	2.16	3.002 (4)	169
O6—H6*A*⋯O4^ii^	0.85	1.78	2.628 (4)	174
O6—H6*B*⋯O7^iii^	0.85	1.87	2.677 (4)	156
O7—H7*B*⋯O2^iv^	0.85	2.04	2.827 (4)	153
O7—H7*C*⋯O1^v^	0.85	2.25	3.029 (4)	153

Symmetry codes: (i) 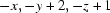; (ii) 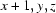; (iii) 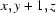; (iv) 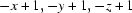; (v) 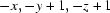.

**Fig. 1.**
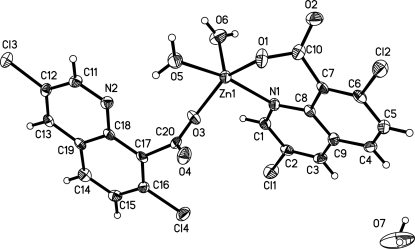
The structure of (I), with the atomic numbering scheme and displacement ellipsoids at the 50% probability level. All H atoms have been omitted for clarity.

**Fig. 2.**
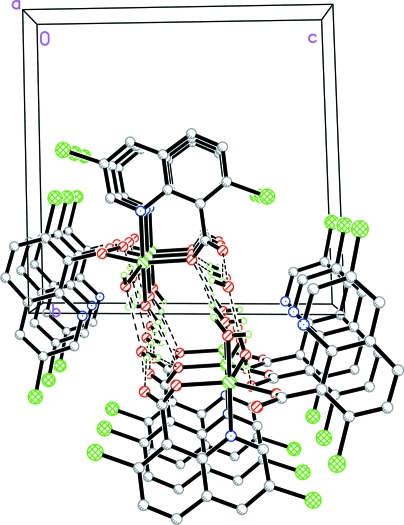
Part of the crystal structure of (I), showing the 1-D helical chain.H atoms bonded to C atoms have been omitted for clarity.

## supplementary crystallographic information

### Comment

Quinolinecarboxylate derivatives and their complexes have attracted considerable interest, because of their interesting high germicidal, antitumoral and pharmacological properties (Adnan *et al.*, 2003; Lumme *et al.*, 1984). Although some transition metal complexes of quinolinecarboxylate ligands have been reported (Che *et al.*, 2005; Chen *et al.*, 2001; Yang *et al.*, 2005), the crystal structures of the 3,7-Dichloro-8-quinolinecarboxylate and its complexes are limited in number, the only two examples are [*M*(C_10_H_4_Cl_2_NO_2_)_2_]_n_(*M* = Ni, Co) (Li *et al.*, 2008; Zhang *et al.*, 2007). we report herein on the structure of [Zn(C_10_H_4_Cl_2_NO_2_)_2_(H_2_O)_2_].H_2_O, (I).

The title complex, (I) crystallizes in the triclinic space group *P*1, with free water molecules in the crystal structure.The Zn(II) center exhibits a distorted square-pyramidal geometry defined by two O atoms and one N atom from two distinct 3,7-Dichloro-8-quinolinecarboxylate ligands, and two water molecules (Fig.1). The N1, O1, O3 and O5 atoms form the base plane, while the O6 atom occupies the axial position.The carboxylate group is bound in a monodentate fashion, with a weak intramolecular O—H···O H-bond between the carboxylate O atom and water molecules. The [Zn(C_10_H_4_Cl_2_NO_2_)_2_(H_2_O)_2_] molecules are linked *via* these H-bond interactions into a 1-D helical chain along the [100] direction (Fig. 2).

### Experimental

The source materials of Zinc hydroxide (0.005 g) and Quinclorac (3,7-Dichloro-8-quinolinecarboxylic acid) (0.024 g) dissolved in 10 ml distilled water and were carefully mixed, and then loaded into a Teflon-lined stainless steel autoclave. The sealed autoclave was heated to 433 K and maintained at this temperature for 48 h. After cooling to room temperature, then some colorless column crystal was obtained.

### Refinement

All H atoms were positioned geometrically and were allowed to ride on their parent atoms.

### Crystal data

**Table:**

[Zn(C_10_H_4_Cl_2_NO_2_)_2_(H_2_O)_2_]·H_2_O	*Z* = 2
*M**_r_* = 601.50	*F*_000_ = 604
Triclinic, *P*1	*D*_x_ = 1.802 Mg m^−^^3^
Hall symbol: -P 1	Mo *K*α radiation λ = 0.71073 Å
*a* = 6.8678 (3) Å	Cell parameters from 1973 reflections
*b* = 12.6996 (5) Å	θ = 1.6–26.0º
*c* = 12.9317 (5) Å	µ = 1.64 mm^−^^1^
α = 87.572 (1)º	*T* = 296 (2) K
β = 82.893 (1)º	Column, colourless
γ = 82.255 (1)º	0.21 × 0.17 × 0.15 mm
*V* = 1108.63 (8) Å^3^	

### Data collection

**Table:**

Rigaku Mercury diffractometer	4308 independent reflections
Radiation source: fine-focus sealed tube	3099 reflections with *I* > 2σ(*I*)
Monochromator: graphite	*R*_int_ = 0.080
Detector resolution: 7.31 pixels mm^-1^	θ_max_ = 26.0º
*T* = 296(2) K	θ_min_ = 1.6º
ω scans	*h* = −8→8
Absorption correction: multi-scan(CrystalClear; Rigaku/MSC, 2001)	*k* = −15→15
*T*_min_ = 0.725, *T*_max_ = 0.791	*l* = −15→14
14022 measured reflections	

### Refinement

**Table:**

Refinement on *F*^2^	Secondary atom site location: difference Fourier map
Least-squares matrix: full	Hydrogen site location: inferred from neighbouring sites
*R*[*F*^2^ > 2σ(*F*^2^)] = 0.041	H-atom parameters constrained
*wR*(*F*^2^) = 0.094	*w* = 1/[σ^2^(*F*_o_^2^) + (0.0408*P*)^2^] where *P* = (*F*_o_^2^ + 2*F*_c_^2^)/3
*S* = 0.97	(Δ/σ)_max_ = 0.001
4308 reflections	Δρ_max_ = 1.20 e Å^−^^3^
307 parameters	Δρ_min_ = −0.80 e Å^−^^3^
Primary atom site location: structure-invariant direct methods	Extinction correction: none

### Special details

**Table:**

Geometry. All e.s.d.'s (except the e.s.d. in the dihedral angle between two l.s. planes) are estimated using the full covariance matrix. The cell e.s.d.'s are taken into account individually in the estimation of e.s.d.'s in distances, angles and torsion angles; correlations between e.s.d.'s in cell parameters are only used when they are defined by crystal symmetry. An approximate (isotropic) treatment of cell e.s.d.'s is used for estimating e.s.d.'s involving l.s. planes.
Refinement. Refinement of *F*^2^ against ALL reflections. The weighted *R*-factor *wR* and goodness of fit *S* are based on *F*^2^, conventional *R*-factors *R* are based on *F*, with *F* set to zero for negative *F*^2^. The threshold expression of *F*^2^ > σ(*F*^2^) is used only for calculating *R*-factors(gt) *etc*. and is not relevant to the choice of reflections for refinement. *R*-factors based on *F*^2^ are statistically about twice as large as those based on *F*, and *R*- factors based on ALL data will be even larger.

### Fractional atomic coordinates and isotropic or equivalent isotropic displacement parameters (Å^2^)

**Table:**

	*x*	*y*	*z*	*U*_iso_*/*U*_eq_	
C1	0.2965 (5)	0.6256 (3)	0.2794 (3)	0.0197 (8)	
H1	0.3070	0.6713	0.2215	0.024*	
C2	0.3295 (5)	0.5161 (3)	0.2636 (3)	0.0194 (8)	
C3	0.3176 (5)	0.4475 (3)	0.3464 (3)	0.0209 (8)	
H3	0.3380	0.3746	0.3366	0.025*	
C4	0.2565 (5)	0.4215 (3)	0.5377 (3)	0.0210 (8)	
H4	0.2778	0.3480	0.5315	0.025*	
C5	0.2088 (5)	0.4641 (3)	0.6336 (3)	0.0241 (9)	
H5	0.1921	0.4201	0.6925	0.029*	
C6	0.1848 (5)	0.5751 (3)	0.6434 (3)	0.0186 (8)	
C7	0.2042 (5)	0.6439 (3)	0.5590 (3)	0.0176 (8)	
C8	0.2434 (5)	0.5998 (3)	0.4583 (3)	0.0173 (8)	
C9	0.2741 (5)	0.4879 (3)	0.4474 (3)	0.0188 (8)	
C10	0.2017 (6)	0.7613 (3)	0.5715 (3)	0.0220 (8)	
C11	−0.2472 (5)	1.1168 (3)	0.1346 (3)	0.0194 (8)	
H11	−0.2486	1.1675	0.1846	0.023*	
C12	−0.2620 (5)	1.1520 (3)	0.0307 (3)	0.0182 (8)	
C13	−0.2648 (5)	1.0809 (3)	−0.0443 (3)	0.0179 (8)	
H13	−0.2756	1.1034	−0.1129	0.022*	
C14	−0.2534 (5)	0.8915 (3)	−0.0880 (3)	0.0221 (8)	
H14	−0.2643	0.9098	−0.1577	0.027*	
C15	−0.2399 (5)	0.7872 (3)	−0.0559 (3)	0.0213 (8)	
H15	−0.2427	0.7346	−0.1034	0.026*	
C16	−0.2218 (5)	0.7597 (3)	0.0488 (3)	0.0196 (8)	
C17	−0.2162 (5)	0.8341 (3)	0.1213 (3)	0.0167 (8)	
C18	−0.2329 (5)	0.9426 (3)	0.0898 (3)	0.0170 (8)	
C19	−0.2509 (5)	0.9721 (3)	−0.0157 (3)	0.0172 (8)	
C20	−0.1707 (5)	0.8066 (3)	0.2315 (3)	0.0179 (8)	
Cl1	0.37704 (15)	0.47230 (7)	0.13739 (7)	0.0300 (2)	
Cl2	0.12831 (15)	0.62268 (8)	0.76860 (7)	0.0300 (2)	
Cl3	−0.28261 (14)	1.28675 (7)	0.00156 (7)	0.0247 (2)	
Cl4	−0.19519 (15)	0.62565 (7)	0.08517 (8)	0.0295 (2)	
N1	0.2514 (4)	0.6674 (2)	0.3722 (2)	0.0185 (7)	
N2	−0.2316 (4)	1.0165 (2)	0.1644 (2)	0.0190 (7)	
O1	0.0705 (4)	0.82485 (19)	0.52664 (19)	0.0294 (7)	
O2	0.3222 (4)	0.7909 (2)	0.6231 (2)	0.0325 (7)	
O3	0.0098 (3)	0.80624 (19)	0.24133 (19)	0.0222 (6)	
O4	−0.3021 (4)	0.78885 (19)	0.3020 (2)	0.0289 (6)	
O5	−0.0852 (4)	0.9774 (2)	0.3738 (2)	0.0336 (7)	
H5A	−0.0800	1.0295	0.4126	0.040*	
H5B	−0.1398	0.9842	0.3175	0.040*	
O6	0.3458 (4)	0.9015 (2)	0.3214 (2)	0.0350 (7)	
H6A	0.4589	0.8647	0.3195	0.042*	
H6B	0.3090	0.9657	0.3391	0.042*	
O7	0.3413 (5)	0.1059 (3)	0.3655 (3)	0.0858 (14)	
H7B	0.4519	0.1189	0.3815	0.103*	
H7C	0.2491	0.1273	0.4129	0.103*	
Zn1	0.10699 (6)	0.83389 (3)	0.37255 (3)	0.01835 (13)	

### Atomic displacement parameters (Å^2^)

**Table:**

	*U*^11^	*U*^22^	*U*^33^	*U*^12^	*U*^13^	*U*^23^
C1	0.020 (2)	0.0198 (19)	0.019 (2)	0.0004 (15)	−0.0046 (16)	−0.0023 (16)
C2	0.0166 (19)	0.022 (2)	0.020 (2)	−0.0014 (15)	−0.0030 (15)	−0.0076 (15)
C3	0.020 (2)	0.0158 (19)	0.027 (2)	−0.0019 (15)	−0.0032 (16)	−0.0063 (16)
C4	0.019 (2)	0.0162 (18)	0.029 (2)	−0.0035 (15)	−0.0068 (17)	−0.0012 (16)
C5	0.024 (2)	0.023 (2)	0.026 (2)	−0.0050 (16)	−0.0072 (17)	0.0055 (17)
C6	0.0165 (19)	0.026 (2)	0.0133 (19)	−0.0017 (15)	−0.0024 (15)	−0.0039 (15)
C7	0.0130 (18)	0.0215 (19)	0.019 (2)	0.0014 (15)	−0.0071 (15)	−0.0027 (15)
C8	0.0145 (18)	0.0200 (19)	0.017 (2)	−0.0006 (15)	−0.0037 (15)	−0.0004 (15)
C9	0.0177 (19)	0.0184 (19)	0.019 (2)	0.0008 (15)	−0.0025 (15)	−0.0004 (15)
C10	0.032 (2)	0.0191 (19)	0.0123 (19)	0.0004 (17)	0.0038 (17)	−0.0019 (15)
C11	0.020 (2)	0.0167 (19)	0.021 (2)	0.0004 (15)	−0.0006 (16)	−0.0068 (15)
C12	0.0152 (18)	0.0160 (18)	0.022 (2)	0.0006 (15)	0.0008 (15)	0.0017 (15)
C13	0.0128 (18)	0.0232 (19)	0.018 (2)	−0.0009 (15)	−0.0041 (15)	0.0017 (16)
C14	0.023 (2)	0.029 (2)	0.014 (2)	−0.0046 (17)	0.0010 (16)	0.0006 (16)
C15	0.0192 (19)	0.022 (2)	0.023 (2)	−0.0018 (16)	−0.0013 (16)	−0.0082 (16)
C16	0.0161 (19)	0.0153 (18)	0.028 (2)	−0.0011 (15)	−0.0049 (16)	0.0000 (16)
C17	0.0068 (17)	0.0229 (19)	0.021 (2)	−0.0022 (14)	−0.0032 (14)	0.0007 (15)
C18	0.0106 (17)	0.0219 (19)	0.0188 (19)	−0.0006 (14)	−0.0037 (15)	−0.0023 (15)
C19	0.0087 (17)	0.0237 (19)	0.019 (2)	−0.0008 (15)	−0.0023 (14)	−0.0016 (15)
C20	0.022 (2)	0.0102 (17)	0.021 (2)	0.0001 (15)	−0.0038 (16)	−0.0012 (15)
Cl1	0.0431 (6)	0.0251 (5)	0.0207 (5)	−0.0030 (4)	0.0021 (4)	−0.0090 (4)
Cl2	0.0445 (6)	0.0278 (5)	0.0170 (5)	−0.0053 (4)	−0.0006 (4)	−0.0009 (4)
Cl3	0.0300 (5)	0.0170 (5)	0.0251 (5)	0.0009 (4)	−0.0006 (4)	0.0019 (4)
Cl4	0.0426 (6)	0.0176 (5)	0.0297 (6)	−0.0056 (4)	−0.0077 (5)	−0.0008 (4)
N1	0.0214 (16)	0.0166 (15)	0.0173 (17)	0.0003 (13)	−0.0044 (13)	−0.0005 (13)
N2	0.0186 (16)	0.0175 (16)	0.0202 (17)	−0.0007 (13)	−0.0011 (13)	−0.0023 (13)
O1	0.0448 (18)	0.0228 (14)	0.0167 (14)	0.0110 (13)	−0.0039 (13)	−0.0015 (11)
O2	0.0441 (18)	0.0280 (15)	0.0284 (16)	−0.0120 (13)	−0.0078 (14)	−0.0041 (12)
O3	0.0171 (14)	0.0299 (14)	0.0196 (14)	0.0019 (11)	−0.0063 (11)	−0.0047 (11)
O4	0.0284 (15)	0.0272 (15)	0.0289 (16)	−0.0032 (12)	0.0019 (13)	0.0085 (12)
O5	0.0471 (18)	0.0223 (14)	0.0319 (17)	0.0073 (13)	−0.0161 (14)	−0.0108 (12)
O6	0.0202 (15)	0.0234 (15)	0.060 (2)	−0.0003 (12)	−0.0006 (14)	−0.0041 (13)
O7	0.050 (2)	0.075 (3)	0.132 (4)	−0.030 (2)	0.031 (2)	−0.055 (3)
Zn1	0.0206 (2)	0.0172 (2)	0.0168 (2)	−0.00023 (17)	−0.00297 (17)	−0.00061 (17)

### Geometric parameters (Å, °)

**Table:**

C1—N1	1.316 (4)	C13—H13	0.9300
C1—C2	1.399 (5)	C14—C15	1.365 (5)
C1—H1	0.9300	C14—C19	1.419 (5)
C2—C3	1.352 (5)	C14—H14	0.9300
C2—Cl1	1.725 (4)	C15—C16	1.400 (5)
C3—C9	1.407 (5)	C15—H15	0.9300
C3—H3	0.9300	C16—C17	1.366 (5)
C4—C5	1.359 (5)	C16—Cl4	1.739 (3)
C4—C9	1.414 (5)	C17—C18	1.414 (5)
C4—H4	0.9300	C17—C20	1.514 (5)
C5—C6	1.406 (5)	C18—N2	1.376 (4)
C5—H5	0.9300	C18—C19	1.415 (5)
C6—C7	1.374 (5)	C20—O4	1.237 (4)
C6—Cl2	1.732 (4)	C20—O3	1.261 (4)
C7—C8	1.421 (5)	N1—Zn1	2.211 (3)
C7—C10	1.504 (5)	O1—Zn1	1.978 (2)
C8—N1	1.377 (4)	O3—Zn1	1.960 (2)
C8—C9	1.419 (5)	O5—Zn1	2.101 (2)
C10—O2	1.231 (4)	O5—H5A	0.85
C10—O1	1.301 (4)	O5—H5B	0.86
C11—N2	1.310 (4)	O6—Zn1	1.982 (2)
C11—C12	1.410 (5)	O6—H6A	0.85
C11—H11	0.9300	O6—H6B	0.85
C12—C13	1.356 (5)	O7—H7B	0.85
C12—Cl3	1.727 (3)	O7—H7C	0.85
C13—C19	1.409 (5)		
			
N1—C1—C2	123.4 (3)	C14—C15—C16	119.8 (3)
N1—C1—H1	118.3	C14—C15—H15	120.1
C2—C1—H1	118.3	C16—C15—H15	120.1
C3—C2—C1	119.8 (3)	C17—C16—C15	122.2 (3)
C3—C2—Cl1	121.7 (3)	C17—C16—Cl4	119.5 (3)
C1—C2—Cl1	118.5 (3)	C15—C16—Cl4	118.2 (3)
C2—C3—C9	119.2 (3)	C16—C17—C18	118.8 (3)
C2—C3—H3	120.4	C16—C17—C20	123.6 (3)
C9—C3—H3	120.4	C18—C17—C20	117.3 (3)
C5—C4—C9	120.5 (3)	N2—C18—C17	118.0 (3)
C5—C4—H4	119.7	N2—C18—C19	122.2 (3)
C9—C4—H4	119.7	C17—C18—C19	119.9 (3)
C4—C5—C6	119.7 (3)	C13—C19—C18	118.2 (3)
C4—C5—H5	120.1	C13—C19—C14	122.8 (3)
C6—C5—H5	120.1	C18—C19—C14	119.0 (3)
C7—C6—C5	122.6 (3)	O4—C20—O3	125.9 (3)
C7—C6—Cl2	120.7 (3)	O4—C20—C17	121.5 (3)
C5—C6—Cl2	116.7 (3)	O3—C20—C17	112.6 (3)
C6—C7—C8	117.8 (3)	C1—N1—C8	118.3 (3)
C6—C7—C10	121.9 (3)	C1—N1—Zn1	115.1 (2)
C8—C7—C10	120.1 (3)	C8—N1—Zn1	123.8 (2)
N1—C8—C9	121.0 (3)	C11—N2—C18	117.6 (3)
N1—C8—C7	118.9 (3)	C10—O1—Zn1	117.0 (2)
C9—C8—C7	120.1 (3)	C20—O3—Zn1	123.7 (2)
C3—C9—C4	122.6 (3)	Zn1—O5—H5A	124.5
C3—C9—C8	118.3 (3)	Zn1—O5—H5B	109.3
C4—C9—C8	119.1 (3)	H5A—O5—H5B	123.2
O2—C10—O1	124.4 (3)	Zn1—O6—H6A	119.1
O2—C10—C7	118.4 (3)	Zn1—O6—H6B	101.5
O1—C10—C7	117.2 (3)	H6A—O6—H6B	129.9
N2—C11—C12	123.4 (3)	H7B—O7—H7C	110.1
N2—C11—H11	118.3	O3—Zn1—O1	147.92 (11)
C12—C11—H11	118.3	O3—Zn1—O6	101.43 (11)
C13—C12—C11	120.2 (3)	O1—Zn1—O6	110.65 (12)
C13—C12—Cl3	120.9 (3)	O3—Zn1—O5	86.43 (10)
C11—C12—Cl3	118.9 (3)	O1—Zn1—O5	90.82 (10)
C12—C13—C19	118.4 (3)	O6—Zn1—O5	94.10 (11)
C12—C13—H13	120.8	O3—Zn1—N1	87.67 (10)
C19—C13—H13	120.8	O1—Zn1—N1	88.61 (10)
C15—C14—C19	120.3 (3)	O6—Zn1—N1	97.37 (11)
C15—C14—H14	119.8	O5—Zn1—N1	167.94 (11)
C19—C14—H14	119.8		

### Hydrogen-bond geometry (Å, °)

**Table:**

*D*—H···*A*	*D*—H	H···*A*	*D*···*A*	*D*—H···*A*
O5—H5A···O1^i^	0.85	2.05	2.889 (4)	166
O5—H5B···N2	0.86	2.16	3.002 (4)	169
O6—H6A···O4^ii^	0.85	1.78	2.628 (4)	174
O6—H6B···O7^iii^	0.85	1.87	2.677 (4)	156
O7—H7B···O2^iv^	0.85	2.04	2.827 (4)	153
O7—H7C···O1^v^	0.85	2.25	3.029 (4)	153

Symmetry codes: (i) −*x*, −*y*+2, −*z*+1; (ii) *x*+1, *y*, *z*; (iii) *x*, *y*+1, *z*; (iv) −*x*+1, −*y*+1, −*z*+1; (v) −*x*, −*y*+1, −*z*+1.
